# Neuroligins and Neurodevelopmental Disorders: X-Linked Genetics

**DOI:** 10.3389/fnsyn.2020.00033

**Published:** 2020-08-11

**Authors:** Thien A. Nguyen, Alexander W. Lehr, Katherine W. Roche

**Affiliations:** ^1^Receptor Biology Section, National Institute of Neurological Disorders and Stroke, National Institutes of Health, Bethesda, MD, United States; ^2^Department of Pharmacology and Physiology, Georgetown University, Washington, DC, United States

**Keywords:** autism, intellectual disabililties, NLGN3, NLGN4X, neuroligin

## Abstract

Autism spectrum disorder (ASD) is a neurodevelopmental disorder that results in social-communication impairments, as well as restricted and repetitive behaviors. Moreover, ASD is more prevalent in males, with a male to female ratio of 4 to 1. Although the underlying etiology of ASD is generally unknown, recent advances in genome sequencing have facilitated the identification of a host of associated genes. Among these, synaptic proteins such as cell adhesion molecules have been strongly linked with ASD. Interestingly, many large genome sequencing studies exclude sex chromosomes, which leads to a shift in focus toward autosomal genes as targets for ASD research. However, there are many genes on the X chromosome that encode synaptic proteins, including strong candidate genes. Here, we review findings regarding two members of the neuroligin (NLGN) family of postsynaptic adhesion molecules, *NLGN3* and *NLGN4*. Neuroligins have multiple isoforms (NLGN1-4), which are both autosomal and sex-linked. The sex-linked genes, *NLGN3* and *NLGN4*, are both on the X chromosome and were among the first few genes to be linked with ASD and intellectual disability (ID). In addition, there is a less studied human neuroligin on the Y chromosome, NLGN4Y, which forms an X-Y pair with NLGN4X. We will discuss recent findings of these neuroligin isoforms regarding function at the synapse in both rodent models and human-derived differentiated neurons, and highlight the exciting challenges moving forward to a better understanding of ASD/ID.

## Introduction

Autism spectrum disorder (ASD) is a highly prevalent neurodevelopmental disorder affecting one in 54 children in the United States. ASD is characterized by deficits in communication and social interaction (Miles, 2011; Fombonne, 2013). Intellectual disability (ID) is characterized by deficits in intellectual functioning and adaptive behavior thus limiting an individual’s ability to thrive independently (Raymond, 2006; Lubs et al., 2012; Ellison et al., 2013). Interestingly, both ASD and ID are more prevalent in males (Geschwind, 2011; Miles, 2011; Werling and Geschwind, 2013; Werling et al., 2016), although this strong sex bias in ASD remains unclear. It is notable that a subset of ASD-associated genes are located on the X chromosome indicating that the sex chromosomes may play a role in at least some of the sexual dimorphism in these disorders.

Autism spectrum disorder is divided into two categories: syndromic and nonsyndromic. Syndromic ASD is defined as a condition in patients who already have an existing neurological disorder. For example, a subset of patients with Fragile-X syndrome, tuberous sclerosis, or Rett syndrome display phenotypes that are attributed to ASD (Singh and Eroglu, 2013; Geschwind and State, 2015). Nonsyndromic ASD accounts for ASD cases that are not linked to any neurological disorders. Before the advancement of next-generation sequencing (NGS), genetic researchers focused on finding rare genetic variants in ASD and ID pedigrees to link genes to these disorders, which led to the association of the neuroligins NLGN3 and NLGN4X to ASD/ID (Jamain et al., 2003; Laumonnier et al., 2004). Other notable genes identified through rare *de novo* mutations and recessive inheritance mutations include *SHANK3*, *CNTNAP2*, *NRXN1*, *PTEN*, *FMR1*, and *TSC1* (Geschwind and State, 2015). Although these cases are rare, functional and genetic studies definitively showed their link with ASD and ID. With NGS becoming cheaper and easier to access, genome wide association studies (GWAS) and whole exome sequencing (WES) studies became the major approaches used to identify common and rare variants for ASD/ID. Large cohort studies continue to identify more genes associated with ASD/ID, including genes that are important in chromatin modification, transcriptional regulation, or are FMRP-associated, embryonically expressed, or affect synaptic function (Sanders et al., 2012; Yu et al., 2013; De Rubeis et al., 2014; Iossifov et al., 2014). Although NGS has dramatically accelerated the identification of new risk genes, it is important to mention that NGS studies often ignore the sex chromosomes due to the limitations for statistical analysis (Wise et al., 2013; No Author List, 2017).

The neuroligin (NLGN) family of postsynaptic cell adhesion molecules have emerged as important factors regulating neuronal development and synaptic transmission. There are five members of the NLGN family in humans and other primates: NLGN1, 2, 3, 4X, and 4Y (Bemben et al., 2015b; Jeong et al., 2017; Südhof, 2017, 2018). However, in rodents, there are only four members: NLGN1, 2, 3, and 4-like (Bolliger et al., 2001, 2008). NLGNs have an isoform-specific localization: NLGN1 is localized to excitatory synapses, NLGN2 at inhibitory synapses, and NLGN3 is at both (Chih et al., 2005; Chubykin et al., 2007; Bemben et al., 2015b). Interestingly human NLGN4X is localized at excitatory synapses, whereas mouse NLGN4-like is at glycinergic synapses (Hoon et al., 2011; Bemben et al., 2015a; Chanda et al., 2016; Marro et al., 2019). NLGN4X and NLGN4Y were historically grouped together and assumed to have the same function due to their almost identical sequence identity. However, recent findings show that a single amino acid difference in NLGN4Y results in a trafficking deficit leading to decreased surface expression and synaptic function (Nguyen et al., 2020).

Neuroligins are highly dynamic, regulated via posttranslational modifications and protein–protein interactions. NLGN1 is phosphorylated by calcium/calmodulin-dependent protein kinase 2 (CaMKII), protein kinase A (PKA), and tyrosine kinases to regulate its function at excitatory synapses (Bemben et al., 2013; Giannone et al., 2013; Letellier et al., 2018; Jeong et al., 2019). Furthermore, a recent paper established that NLGN1-mediated synaptogenic properties are mediated by interacting with Kalirin7, a Rho guanine nucleotide exchange factor (GEF) (Paskus et al., 2019, 2020). Phosphorylation of NLGN2 affects binding with inhibitory scaffolding proteins, thus regulating its function at inhibitory synapses (Poulopoulos et al., 2009; Antonelli et al., 2014; Nguyen et al., 2016). NLGN3 can be cleaved by proteases to reduce its function at synapses (Bemben et al., 2019). Interestingly, the extracellular cleaved fragment of NLGN3 has been identified as a potent mitogen in brain cancer (Venkatesh et al., 2015, 2017). Lastly, NLGN4X can be phosphorylated by protein kinase C (PKC) to enhance excitatory synapses (Bemben et al., 2015a). Together, NLGNs comprise an important class of proteins that are dynamic and have multiple functions at synapses.

Of the NLGN family, *NLGN3*, *NLGN4X*, and *NLGN4Y* are sex-linked with *NLGN3* and *NLGN4X* on the X-chromosome and *NLGN4Y* on the Y-chromosome. Early genetic studies using a family pedigree of ASD/ID probands associated *NLGN3* and *NLGN4X* with ASD/ID (Jamain et al., 2003; Laumonnier et al., 2004) (Tables 1, 2). Interestingly, the majority of cases for NLGN3- and NLGN4X-associated ASD/ID are males. In this review, we provide an overview of the current literature of sex-linked NLGNs functions and their links to ASD/ID.

**TABLE 1:** ASD-associated NLGN3 variants.

Variants	Sex	Inheritance pattern	Primary Phenotype	Additional Comments/References
P104Qfs42X	N/A	N/A	ASD	Kenny et al. (2014)
R195W	N/A	De novo	ASD	Iossifov et al. (2014)
V306M	N/A	Maternal	ASD	Jiang et al. (2013)
V321A	M	Maternal	ASD	Yu et al. (2013)
N390X	N/A	Maternal	ASD	Yuen et al. (2017)
G426S	F	De novo	ASD	Xu et al. (2014)
W433X	M	Maternal	ASD	McRae et al. (2017)
R451C	M	Maternal	ASD	Jamain et al. (2003)
P514S	M × 2	Maternal	ASD	Quartier et al. (2019)
R597W	M × 3	Maternal	ASD	Quartier et al. (2019); Redin et al. (2014)
R617W	M × 2	Maternal	ASD/ID	Redin et al. (2014)
T632A	N/A	Maternal	ASD	Blasi et al. (2006)

**TABLE 2:** ASD-associated NLGN4X variants.

Variants	Inheritance Pattern	Sex	Primary Phenotype	Additional Comments/References
G84R	Maternal	M	ASD	Asymptomatic mothers (Xu et al., 2014)
R87W	De novo	M	ASD	Zhang et al. (2009)
P94L	N/A	N/A	N/A	GeneDX submitted on ClinVar with unknown significance
G99S	Maternal	F	ASD	Mother also has learning disability. A brother also has learning disability (Yan et al., 2005)
		M	ASD	Mother also has learning disability. Sibling of above (Yan et al., 2005)
R101Q	Maternal	M	ASD	Nguyen et al. (2020)
V109L	Maternal	M	ID	Nguyen et al. (2020)
Q162K	De novo	F	ASD	Xu et al. (2014)
L211X	N/A	N/A	Anxiety, ADHD, Cerebral palsy	Yuen et al. (2017)
Q274X	Maternal	M	ADHD	Yuen et al. (2017)
A283T	Maternal	M	ASD	Xu et al. (2014)
Q329X	Maternal	M	ASD	Yu et al. (2013)
K378R	Maternal	M	ASD	Pampanos et al. (2009)
		M	ASD	Yan et al. (2005)
396X frameshift 1186t	Maternal	2 × M	Asperger’s syndrome/ASD	Jamain et al. (2003)
V403M	Maternal	M	ASD	Have both affected and unaffected siblings (Xu et al., 2014)
429X (nt1253del(AG)	Maternal	13 × M	ASD/ID	Laumonnier et al. (2004)
V454_A457X	De novo	M	ID	Martínez et al. (2016)
V522M	De novo	N/A	TD	Wang et al. (2018)
R704C	Maternal	M	ASD	Unaffected sister (+/−) (Yan et al., 2005)
R766Q	Maternal	M	ASD	Yu et al. (2013)

## NLGN3 and ASD

The first link between ASD and *NLGN3* was revealed from a case study of ASD patients. Jamain et al. (2003) identified a missense mutation in a Swedish family with two affected brothers, one with ASD and the other with Asperger’s syndrome. They showed that both probands contain a missense mutation in NLGN3 (NLGN3 R451C), which encodes an arginine instead of a cysteine at amino acid 451 within the extracellular domain (ECD) of NLGN3. The NLGN3 R451C mutant displays a decrease in surface expression compared to WT, and is retained in the ER by binding to the chaperone protein BiP (Chih et al., 2004; Comoletti et al., 2004; De Jaco et al., 2006). Unlike the human specific *NLGN4X*, *NLGN3* is highly conserved across mammals, facilitating the development of knock-in (KI) mouse models to study how NLGN3 mutations affect behavior.

In agreement with molecular studies, the NLGN3 R451C KI mouse displays a significant (∼90%) decrease in protein levels compared to WT. Interestingly, the NLGN3 R451C mutant demonstrated a synaptic transmission gain-of-function phenotype, and these effects are synapse specific. Although the NLGN3 R451C KI mice have reduced protein levels, NLGN3 R451C mice, but not NLGN3 KO mice, display an increase in inhibitory synapses as measured by VGAT and gephyrin immunoreactivity. Furthermore, a concomitant increase in mIPSCs frequency in somatosensory cortex was observed in NLGN3 R451C mice, but not NLGN3 KO mice (Tabuchi et al., 2007). In addition, NLGN3 R451C leads to impaired inhibitory synaptic transmission in PV-neurons in KI animals, unlike the NLGN3 KO; however, both mouse lines show enhanced inhibitory synaptic transmission in cholecystokinin basket cells (Földy et al., 2013). Horn and Nicoll (2018) also provide additional evidence of the synapse-specific function of NLGN3 by showing that knocking down NLGN3 using miRNA specifically affected IPSCs recorded from somatostatin neurons, but not from PV-neurons. In addition, NLGN3 R451C mice, but not NLGN3 KO mice, have a striking phenotype at glutamatergic synapses. In the CA1 region of the hippocampus, NLGN3 R451C mice display an increase in excitatory synaptic transmission (Etherton et al., 2011). Along with this observation, Etherton et al. (2011) saw an increase in dendritic complexity and NMDAR protein levels in NLGN3 R451C mice. In contrast, NLGN3 R451C mice display impaired synaptic transmission at the calyx of Held synapses. Furthermore, Zhang et al. (2017) elegantly demonstrated that the synaptic effect of NLGN3 on the calyx of Held synapses is only observed when NLGN3 is deleted late, but not early, in development. Lastly, NLGN3 R451C KI mice and NLGN3 KO mice share a common synaptic defect at striatal synapses; the deletion or KI of NLGN3 in D1 neurons, but not D2 neurons, results in a decrease in mIPSCs frequency (Rothwell et al., 2014). Taken together, the NLGN3 R451C mutation differentially alter synaptic function depending on neuron and synapse type.

Behavioral analyses of NLGN3 R451C KI mice revealed a deficit in social interaction and an enhancement in spatial learning; however, these findings were not reproduced in a separate independent study, likely due to differences in mouse strains or behavioral protocols (Tabuchi et al., 2007; Chadman et al., 2008; Jaramillo et al., 2014; Lakhani et al., 2019). Another phenotype of ASD is repetitive behavior; and, interestingly, the NLGN3 R451C KI and NLGN3 KO mice share this phenotype despite differences in social interaction and spatial memory paradigms (Rothwell et al., 2014; Burrows et al., 2015). Indeed, NLGN3 R451C KI and NLGN3 KO mice both have an enhanced ability to stay on an accelerated rod (Chadman et al., 2008; Rothwell et al., 2014). Importantly, the repetitive behavior of NLGN3 mutants is due to dysfunction of D1-dopamine receptor-expressing medium spiny neurons, but not D2 neurons. Taken together, the ASD phenotypes of NLGN3 R451C KI and NLGN3 KO mice are circuit- and neuron-specific. Further investigations into which circuits affect the social interaction, spatial memory, and social memory phenotypes in NLGN3 R451C and NLGN3 KO are needed to better understand the mechanisms driving these behavioral deficits in ASD.

Studies in NLGN3 R451C KI and NLGN3 KO mice highlighted a need to better understand the physiological function of NLGN3. For example, a striking observation in NLGN3 R451C KI mice is a ∼90% reduction in protein levels, while displaying both gain-of-function and loss-of-function phenotypes depending on the type of synapses. Different synaptic phenotypes induced by the single point mutation, NLGN3 R451C, suggest that WT NLGN3 normally functions in a context-dependent manner. Indeed, context-dependent function of NLGNs has been reported in which excitatory synapses are regulated by the relative expression of NLGN1. For example, NLGN1 KO mice display similar spine density as WT animals, but when NLGN1 KO neurons are co-cultured with WT neurons, the NLGN1 KO neurons show a reduction in spine density (Kwon et al., 2012). Applying this model of competition to NLGN3 R451C KI mice to explain the gain-of-function observed in this animal is worthy of investigation. It is also important to carefully study NLGN3 function throughout development. Zhang et al. (2017) demonstrated reduced synaptic transmission at the calyx of Held synapse when NLGN3 is deleted late, but not early, in development. They further showed that when NLGN3 is conditionally knocked out in early development, cerebellin-1 can compensate for the lack of NLGN3.

## NLGN4X and Its Link to ASD

### Divergence of NLGN4

Of the ASD-associated genes identified from human genetic screens, NLGNs are of particular interest due to their important function at synapses. Early genetic studies on the X chromosome indicated that a deletion at Xp22.3 was found in ASD/ID probands (Thomas et al., 1999; Zinn et al., 2007). Interestingly, *NLGN4X* is located within this region. Although disease-associated mutations in NLGNs are relatively rare, rigorous genetic studies using probands’ pedigrees have established a causal link between NLGN4X and ASD/ID (Table 2).

**FIGURE 1:**
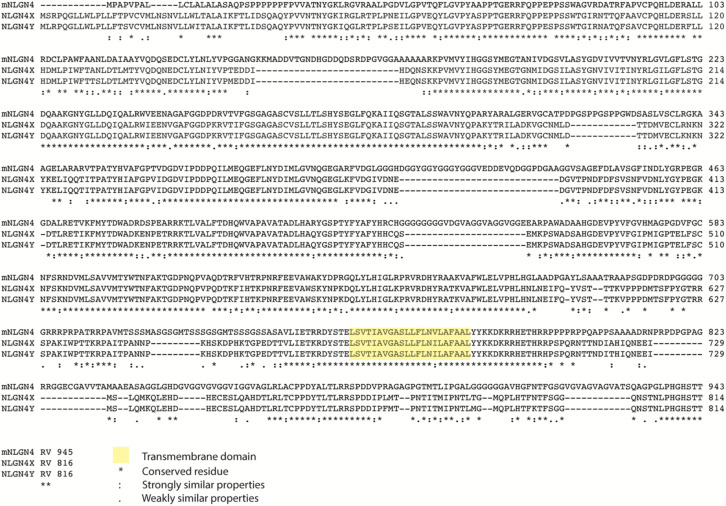
Alignment of NLGN4. Alignment of mouse and human NLGN4s and their conservation.

Because *NLGN4X* is a human-specific gene, the discovery of mouse *NLGN4-like* was exciting because it allowed the study of NLGN4 in rodents to probe its role in ASD/ID. Although, there have been enormous advances in the field regarding the synaptic function of NLGN1-3, there are still many gaps in our understanding of the NLGN4 isoforms, which is complicated due to their unusually rapid divergence in humans and rodents. In humans, NLGN4 is sex-linked, and *NLGN4X* and *NLGN4Y* combine to form an X-Y gene pair. However, in mice, NLGN4 exists as a pseudo-autosomal gene often referred to as NLGN4-like. In addition, Maxeiner et al. (2020) observed that mouse NLGN4-like undergoes rapid evolution resulting in changes in protein sequence. Sequence alignment of NLGN4X with NLGN4-like shows seven insertions in NLGN4-like across both the ECD and intracellular domain (ICD). Interestingly, NLGN4 from the rodent infra-orders *castorimorpha*, *hystricomorpha*, and *sciuromorpha* retains similarity to human NLGN4X, whereas the rodent infra-order *myomorpha*, which includes mice, do not. Thus far NLGN4 has not been identified in rats (Bolliger et al., 2008; Maxeiner et al., 2020). Sequence alignment of mouse NLGN4-like, human NLGN4X, and NLGN4Y shows that NLGN4-like only shares ∼60% sequence identity with NLGN4X/4Y, whereas NLGN4X shares ∼97% sequence identity with NLGN4Y (Figure 1). A decade of research later, it is now clear that the human and rodent NLGN4 genes do not share the same function as previously assumed.

### Human and Mouse NLGN4

Human NLGN4X was first cloned almost two decades ago. In the initial studies, NLGN4X was shown to be expressed and processed in a similar fashion to that of NLGN1. NLGN4X, like NLGN1, is glycosylated, traffics to the cell surface, and can bind to PSD-95 (Bolliger et al., 2001). Furthermore, NLGN4X is found at excitatory synapses. NLGN4X overexpression in mouse hippocampal neurons increases dendritic spine density, but it decreases mEPSCs frequency and amplitude (Chanda et al., 2016; Zhang et al., 2009). However, exogenously expressed human NLGN4X in rat hippocampal slices in combination with NLGN1-3 microRNA to knockdown endogenous NLGN1-3 showed an increase in spine density and a concomitant increase in both AMPAR- and NMDAR-mediated EPSCs (Bemben et al., 2015a). The difference between these two sets of experiments is the presence of endogenous NLGN1-3. It is unclear whether NLGN4X can form heterodimers with NLGN1-3 *in vivo*, although NLGN4X has been shown to form heterodimers with NLGN1 (Poulopoulos et al., 2012). Further investigation into this subject can provide a better understanding of the function of endogenous NLGN4X at synapses.

Using differentiated neurons from human induced pluripotent stem cells (iPS cells), NLGN4X was shown to colocalize with VGLUT and PSD-95, revealing NLGN4X localization at excitatory synapses (Marro et al., 2019). However, in NLGN4X KO differentiated neurons, Marro et al. (2019) did not observe any changes in either EPSCs or IPSCs. It is important to note that although differentiated human neurons from iPS cells can be useful, these differentiated neurons are not fully mature and are lacking NMDARs, a key component of excitatory synapses (Zhang et al., 2013; Quadrato et al., 2017; Marro et al., 2019).

In contrast to NLGN4X, mouse NLGN4-like functions at inhibitory synapses. Localization experiments in mice show that NLGN4-like is at glycinergic inhibitory synapses where it colocalizes with glycine receptors and gephyrin, but not PSD-95 in brainstem, spinal cord, and retina. Moreover, NLGN4-like KO mice were shown to have deficits in glycinergic synaptic transmission (Jamain et al., 2008; Hoon et al., 2011; Zhang et al., 2018). In addition, NLGN4-like also functions at GABAergic synapses (Hammer et al., 2015; Unichenko et al., 2018). In KO NLGN4-like mice, GABAergic synaptic transmission is impaired in hippocampal CA3 region (Hammer et al., 2015). Together, NLGN4-like primarily acts at inhibitory synapses, either glycinergic or GABAergic, whereas human NLGN4X acts at excitatory synapses.

NLGN4-like KO mice were generated over a decade ago and have been characterized extensively. However, the behavioral data have been complicated. For instance, NLGN4-like KO mice were first characterized as having a deficit in social interaction and vocalization (Jamain et al., 2008; El-Kordi et al., 2013; Ju et al., 2014); however, another study using the same NLGN4-like KO mice did not find any deficit in social interaction or vocalization (Ey et al., 2012). Although NLGN4-like KO mice provide insights into how this protein may function at synapses, because human NLGN4X and mouse NLGN4-like are divergent, there should be caution in linking mouse NLGN4-like studies with NLGN4X-associated neurodevelopmental disorders.

Lastly, NLGNs are dynamically regulated through posttranslational modifications (Bemben et al., 2015b; Jeong et al., 2017). Similar to NLGN1 and NLGN2, posttranslational modifications have an important role in regulating NLGN4X function (Bemben et al., 2015b; Jeong et al., 2017). NLGN4X is phosphorylated by PKC at T707 (Bemben et al., 2015a). Unlike CaMKII phosphorylation of NLGN1, PKC phosphorylation of NLGN4X does not increase its trafficking to the surface. However, phosphorylated NLGN4X T707 does lead to increases in spine density and aggregation of the excitatory synapse markers VGLUT and PSD-95 (Bemben et al., 2013; Bemben et al., 2015a). In addition, analyses of the NLGN4X phospho-mimetic mutation, T707D, reveal significant enhancement of both AMPAR and NMDAR EPSCs compared to WT (Bemben et al., 2015a). How phosphorylated NLGN4X is able to increase excitatory synaptic strength will require additional investigation to reveal the precise mechanisms underlying synaptic potentiation. This topic would benefit from techniques that allow the characterization of spatiotemporal dynamics of PKC phosphorylation of NLGN4X *in vivo*. Furthermore, NLGN4X T707 is conserved in mouse NLGN4-like, but it is unclear whether this residue is phosphorylated in mouse NLGN4-like. Would the phosphorylation of this conserved threonine residue in mouse NLGN4-like enhance synaptic transmission as it does in human NLGN4X? Investigation on the mechanism of phosphorylation and the enhancement of synaptic transmission is a worthy topic to study.

## NLGN4X and ASD/ID

Jamain et al. (2003) first established NLGN4X as causative genes for ASD/ID through screening patients with ASD and Asperger’s syndrome, and identified a frameshift mutation (1186insT) in *NLGN4X*, which leads to a premature stop codon at amino acid 396. Interestingly, in addition to the two probands, their mother also carries the mutation, but does not display any autistic symptoms (Jamain et al., 2003). The most convincing case for *NLGN4X* as an ASD/ID risk gene is from a study following a French family with a nonsense mutation in *NLGN4X*. Laumonnier et al. (2004) observed a 2-base-pair deletion in *NLGN4X* that resulted in a stop codon at position 429. By documenting the clinical data from this large family, Laumonnier et al. (2004) observed that 13 males with the nonsense mutation were diagnosed with ASD, ID, or pervasive neurodevelopmental disorders, whereas female carriers were unaffected. This finding is remarkable in showing that this mutation in *NLGN4X* follows an X-linked recessive pattern. Many subsequent studies have linked *NLGN4X* with neurodevelopmental disorders, and the recurrent theme is that the majority of affected probands are males (Table 2).

Along with frameshift and nonsense mutations, many disease-associated missense mutations have been identified in *NLGN4X*. How might these missense mutations affect NLGN4X function? A missense mutation was identified in two ASD probands resulting in a substitution of an arginine residue to tryptophan (NLGN4X R87W). The NLGN4X R87W variant displays a profound deficit in NLGN4X surface expression, which leads to hypofunction of the protein due to decreased synaptogenesis. Furthermore, expression of NLGN4X R87W results in increased synaptic strength when overexpressed in neurons on a WT background (Zhang et al., 2009). It is puzzling why a variant that failed to induce synaptogenesis on a null background can still enhance synaptic function. Interestingly, a cluster of NLGN4X-associated variants has been identified near the NLGN4X R87W that also display a deficit in surface expression (Nguyen et al., 2020). Because these NLGN4X-associated variants are in the ECD, it is of interest to investigate their ability to bind to neurexin. Using the solved structure of NLGN4X, it was shown that ASD-associated mutations, such as NLGN4X G99S, are located outside of the neurexin binding domain (Fabrichny et al., 2007). These data suggest the observed phenotype from the cluster of NLGN4X-associated mutations is due to a deficit in trafficking.

Another NLGN4X rare variant that has garnered much attention is a substitution in the ICD from arginine to cysteine, NLGN4X R704C (Yan et al., 2005). As discussed above, NLGN4X is phosphorylated by PKC at T707 resulting in an increase in spine numbers and EPSCs (Bemben et al., 2015a). Interestingly, there were significant deficits in phosphorylation of NLGN4X T707 in the NLGN4X R704C variant, and the effects mediated by phosphorylation were abolished (Bemben et al., 2015a). In a separate study, Chanda et al. (2016) expressed NLGN4X R704C in cultured mouse neurons on a WT background and observed an increase in both NMDAR and AMPAR EPSCs compared to WT. Interestingly, neither study observed a change in surface trafficking. The discrepancy in these studies likely results from differences in experimental design, chiefly whether to include or exclude endogenous NLGN1-3. Taken together, NLGN4X R704C displays profound differences, compared to WT, in regulation of excitatory synapses. Using human differentiated neurons from NLGN4X R704C KI hiPSCs, Marro et al. (2019) observed an increase in EPSCs compared to WT. In addition, NLGN4X R704C was shown to increase binding with GluA1, but not PSD-95 (Marro et al., 2019), again revealing that this rare variant has multiple functional effects.

With the advances in stem cell research, it is now possible to study how different NLGN4X variants function in human neurons. Although studies taking this approach provide attractive new tools to study endogenous NLGN4X and its variants, there are pitfalls that needs to be addressed. Use of differentiated neurons from hiPSCs is still in its infancy and synaptic activity from these neurons does not represent the full endogenous nature of a synapse. For instance, it has been shown that differentiated neurons using single transcription expression models lack NMDA receptors (Zhang et al., 2013; Quadrato et al., 2017; Wang et al., 2017; Nehme et al., 2018). These neurons can express NMDARs if, and only if, they are allowed to grow for a long period of time (35+ days). Even so, to date, there is little biochemical evidence that NMDARs are present under these differentiation protocols. For the study of neuroligins, this is particularly problematic as they have been shown to directly interact with NMDARs via their ECDs (Budreck et al., 2013). Thus, although stem cell and differentiation technology are attractive and can be a powerful tool to study human neurons and diseases, a better understanding of the PSD in these neurons is needed before it can be used with great confidence as a model to study synaptic transmission.

## NLGN4X and NLGN4Y

Until recently, the studies on human specific NLGN4s have focused on NLGN4X. However, it is important to explore the function of NLGN4Y as well. NLGN4X and NLGN4Y are remarkably conserved with only 19 amino acid differences between them. Due to this high sequence conservation, the two proteins have been assumed to have the same function (Bemben et al., 2015b; Südhof, 2018); however, this hypothesis had not been experimentally examined until recently. Because NLGN4X/Y are sex-linked genes, an important consideration is the sex-bias in the expression of NLGN4X. Outside of the pseudo autosomal regions (PARs), some genes on the X chromosome can escape X-inactivation thus providing an imbalance of gene dosage between males and females (Carrel and Willard, 2005; Skuse, 2005; Helena Mangs and Morris, 2007; Tukiainen et al., 2017). Interestingly, there are Y-linked genes that are homologs to X-linked genes that escaped X-inactivation in order to balance the gene dosage in males. Furthermore, these X-Y gene pairs have been shown to have an important function in transcription, translation and protein stability (Bellott et al., 2014; Cortez et al., 2014; Hughes and Page, 2015). Together, these studies reveal an important role for genes on the Y chromosome other than sex determining genes. Indeed, comparison of *NLGN4X* and *NLGN4Y* expression in males and females reveals interesting differences. In a large transcriptomic study, *NLGN4Y* was shown to express only in males, as expected; however, *NLGN4X* was shown to express at similar level between males and females (Kang et al., 2011; Trabzuni et al., 2013). To complicate the issue further, a separate study reported that incomplete X-inactivation exists in mammals, and *NLGN4X* partially escapes (Carrel and Willard, 2005; Berletch et al., 2011). Interestingly, in a study using different tissues to study X-inactivation, *NLGN4X* expression is higher in the cortex in female vs. male (Tukiainen et al., 2017). Although gene expression of *NLGN4X* and *NLGN4Y* has been compared, research comparing NLGN4X and NLGN4Y protein function has lagged behind.

Although it was reasonable to hypothesize that NLGN4X and NLGN4Y served the same function due to their high sequence homology (97%), this hypothesis had never been tested. Interestingly, many ASD/ID variants have been identified in NLGN4X (Jamain et al., 2003; Laumonnier et al., 2004; Yan et al., 2005; Volaki et al., 2013; Xu et al., 2014; Bemben et al., 2015a; Chanda et al., 2016), whereas only one missense mutation has been identified in NLGN4Y (Yan et al., 2008). Furthermore, ASD/ID-associated mutations in NLGN4X selectively affect more males than females, and the reason for this male bias is unknown. This strong male bias observation in NLGN4X-associated diseases, prompted us to focus on NLGN4Y. If NLGN4Y and NLGN4X are functionally redundant, then there should not be a male bias in NLGN4X-associated diseases.

**FIGURE 2:**
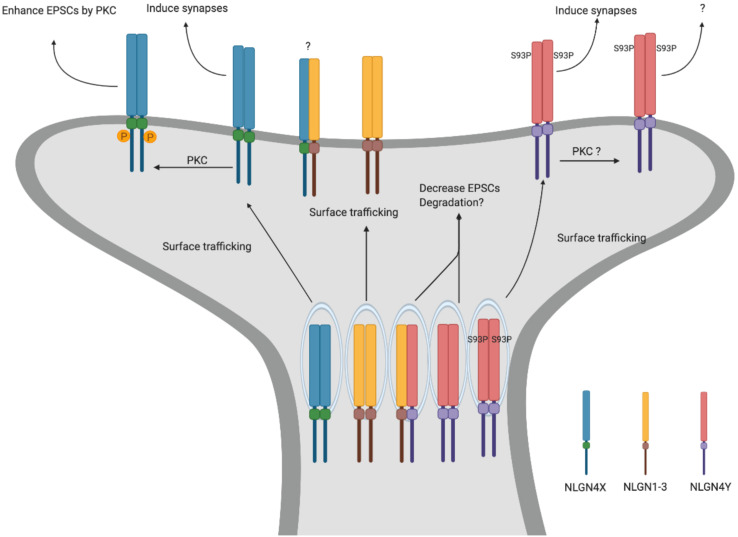
NLGN4X and NLGN4Y function. Schematic for differential trafficking of NLGN4X vs NLGN4Y. NLGN4X can traffic to the surface and induce excitatory synapses. Furthermore, phosphorylation of NLGN4X by PKC drastically enhances excitatory postsynaptic currents (EPSCs). In contrast, NLGN4Y cannot traffic to the surface, thus decreasing EPSCs through binding with other NLGNs.

To explore the function of NLGN4Y, in a recent study, we directly compared NLGN4X and NLGN4Y and found that NLGN4Y cannot traffic to the surface to induce synapses (Nguyen et al., 2020). Furthermore, the differential trafficking observed between NLGN4X and NLGN4Y is due to an amino acid difference at position 93, with proline for NLGN4X and serine for NLGN4Y. Indeed, the NLGN4Y S93P mutant was able to efficiently traffic to the surface and induce synapses. Interestingly, there is a cluster of disease-associated NLGN4X variants surrounding the critical amino acid in NLGN4X. Upon analysis, these variants phenocopied the NLGN4Y trafficking deficit and cannot induce synapses (Figure 2).

What is the function of NLGN4Y if it cannot get to the surface? Nguyen et al. (2020) demonstrated that NLGN4Y can oligomerize with NLGN1, 2, 3, and 4X and reduce their surface trafficking. In addition, exogenously expressed NLGN4Y on a WT background decreased mEPSCs suggesting NLGN4Y acts to inhibit NLGN1-3 function. However, this study relies on exogenously expressed NLGNs in heterologous cells or rat hippocampal neurons. What the role is for endogenous NLGN4Y in human neurons is an important lingering question.

## Conclusion

With the advances in NGS technologies, a wide variety of genes have been associated with ASD/ID. However, many of these studies have ignored the sex chromosomes due to the additional expense and a lack of statistical power. However, historically many genes on the X-chromosome have been linked to ASD/ID by evaluating proband pedigrees. NLGN3 and NLGN4X, both on the X chromosome, were among the first genes associated with ASD/ID. Although NLGN3 and NLGN4X variants only occur in a small population of ASD/ID cases, studies using NLGN3 and NLGN4 mouse models have provided many insights into how disruptions in NLGN3 and NLGN4 function contribute to ASD/ID phenotypes. With advances in stem cell and neuronal differentiation, it is now possible to study NLGN3 and NLGN4X variants using human iPSCs to explore the causality between disruption in sex-linked NLGNs and ASD/ID by examining endogenous human neuroligins. Although neuronal differentiation is an exciting new technology to further our understanding of the human brain, differentiated neurons from human iPSCs are still relatively immature. Further improvement in the technologies to develop reliable mature neurons will be of paramount importance going forward. In addition, the unexpected revelations from the study of NLGN4X and NLGN4Y highlight the need to investigate the often-ignored Y-chromosome. Although many facets of the sex-linked NLGNs have been characterized, many important questions remain unanswered and provide a fertile topic for future investigation into synaptic regulation and to develop therapeutic treatments.

## Author Contributions

TN and KR wrote the manuscript. AL helped to create table for variants.

## Conflict of Interest

The authors declare that the research was conducted in the absence of any commercial or financial relationships that could be construed as a potential conflict of interest.
